# Inhibition of Aflatoxin Production by Citrinin and Non-Enzymatic Formation of a Novel Citrinin-Kojic Acid Adduct

**DOI:** 10.3390/jof9010029

**Published:** 2022-12-23

**Authors:** Masayuki Ichinomiya, Emi Fukushima-Sakuno, Ayaka Kawamoto, Hiroyuki Nakagawa, Hidemi Hatabayashi, Hiromitsu Nakajima, Kimiko Yabe

**Affiliations:** 1 Institute of Food Research, National Agriculture and Food Research Organization (NARO), 2-1-12 Kannon-dai, Ibaraki 305-8642, Japan; 2 Faculty of Agriculture, Tottori University, Koyama, Tottori 680-8553, Japan; 3 Department of Applied Chemistry and Food Science, Fukui University of Technology, 3-6-1 Gakuen, Fukui 910-8505, Japan

**Keywords:** aflatoxin production, *Aspergillus oryzae*, *Aspergillus parasiticus*, CTN-KA adduct, dihydrosterigmatocystin, *Penicillium citrinum*, tip culture method, kojic acid

## Abstract

Screening for microorganisms that inhibit aflatoxin production from environments showed that *Penicillium citrinum* inhibited aflatoxin production by *Aspergillus parasiticus*. The inhibitory substance in the culture medium of *P. citrinum* was confirmed to be citrinin (CTN). RT-PCR analyses showed that CTN did not inhibit expressions of aflatoxin biosynthetic genes (*aflR*, *pksL1*, and *fas-1*) of *A. parasiticus*, whereas feeding experiments using *A. parasiticus* showed that CTN inhibited the in vivo conversion of dihydrosterigmatocystin to AFB_2_·AFG_2_. These results suggest that CTN inhibits a certain post-transcriptional step in aflatoxin biosynthesis. CTN in the culture medium of *A. parasiticus* was found to be decreased or lost with time, suggesting that a certain metabolite produced by *A. parasiticus* is the cause of the CTN decrease; we then purified, characterized, and then analyzed the substance. Physico-chemical analyses confirmed that the metabolite causing a decrease in CTN fluorescence was kojic acid (KA) and the resulting product was identified as a novel substance: (1*R*,3*S*,4*R*)-3,4-dihydro-6,8-dihydroxy-1-(3-hydroxy-6-(hydroxymethyl)-4-oxo-4*H*-pyran-2-yl)-3,4,5-trimethyl-1*H*-isochromene-7-carboxylic acid, which was named “CTN-KA adduct”. Our examination of the metabolites’ toxicities revealed that unlike CTN, the CTN-KA adduct did not inhibit aflatoxin production by *A. parasiticus*. These results indicate that CTN’s toxicity was alleviated with KA by converting CTN to the CTN-KA adduct.

## 1. Introduction

Filamentous fungi produce a variety of secondary metabolites, among which the toxic secondary metabolites are called mycotoxins. Although more than 400 types of mycotoxins have been reported; those most commonly identified in agriculture, livestock and food industries are limited to less than 20 types [1]. Mycotoxin contamination of crops occurs mainly when mycotoxin-producing fungi infect crops during cultivation and storage [1,2]; the ingestion of mycotoxin-contaminated food or feed can result in serious health hazards to livestock and humans. Many countries have set up regulation standards for some mycotoxins in foods [1], and food products that are contaminated with a mycotoxin above the regulated standard value cannot be sold for consumption and are sometimes incinerated. Thus, mycotoxin contamination can cause health problems as well as great economic loss worldwide.

Among the many mycotoxins, aflatoxins (AFs) are known to be the most carcinogenic, teratogenic, and mutagenic, and they are acutely toxic [3,4,5]. *Aspergillus flavus* and *Aspergillus parasiticus* are the major producers of AFs, and some strains of other minor *Aspergillus* species such as *A. nomius*, *A. psudotamarii*, *A. bombycis*, and *A. ochraceoroseus* have been reported to produce AFs [6,7]. Although aflatoxigenic fungi can potentially produce eight types of AFs through complicated biosynthetic pathways, the major AFs produced by fungi are the aflatoxins B_1_ (AFB_1_), B_2_ (AFB_2_), G_1_ (AFG_1_), and G_2_ (AFG_2_) [6,7,8]. Aflatoxin contamination has been reported in crops such as maize, cotton, peanuts, and tree nuts. Various methods to control aflatoxin contamination in crops have been reported, including genetic engineering for crop resistance; biological control with competitive, non-aflatoxigenic strains of *A. flavus*; and reducing AFs or aflatoxin biosynthesis by using fungicides, pesticides, or inhibitory compounds isolated from plant or microbial sources [9,10,11,12,13]. Yan et al. isolated a novel substance that inhibited the production of AFs, i.e., cyclo(L-leucyl-L-prolyl), from the culture medium of *Achromobacter xylosoxidans*, and demonstrated that this substance inhibited a particular step in the transcription of the aflatoxin biosynthetic genes [14]. Iimura et al. reported that cyclo(L-Ala-L-Pro) inhibited the production of AFs and affected the function of glutathione S-transferase in *A. flavus* [15].

Toward the goal of increasing the number of useful microbial candidates that can combat AFs, in the present study, we screened for other microorganisms that are inhibitors of aflatoxin production from various environmental substances such as soils and dead leaves. The results demonstrated that some strains of *Penicillium citrinum* strongly inhibited the production of AFs by *A. parasiticus* and confirmed that the inhibitory substance was citrinin (CTN). CTN is a mycotoxin with a distinctive yellow fluorescence that is known as a nephrotoxic and hepatotoxic substance to animals; it has also shown antibiotic, antifungal, and anti-sarcoma effects on microorganisms, animal cells, and more [16]. CTN is produced by several species that belong to mainly three genera: *Penicillium*, *Monascus*, and *Aspergillus* [16,17,18].

The present findings also demonstrated that the yellow fluorescence of CTN was remarkably decreased in the culture medium of *A. parasiticus*. We confirmed that kojic acid (KA) produced by *A. parasiticus* caused a decrease in the yellow fluorescence of CTN and at the same time produced a novel substance. A physico-chemical method revealed that this substance was composed of CTN and KA through a covalent C-C bond. The substance was named the “CTN-KA adduct” to differentiate it from “substance-KA conjugate”, which refers to binding substances composed of a certain substance and KA through an ester-bond, amido-bond, and so on. The present results also showed that the CTN-KA adduct could be produced non-enzymatically by simply incubating CTN and KA in aqueous conditions. The resulting CTN-KA product did not inhibit aflatoxin production, suggesting that the formation of CTN-KA adduct might have a certain biological function for KA-producing fungi.

## 2. Materials and Methods

### 2.1. Microorganisms

Aflatoxigenic *A. parasiticus* SYS-4 (=NRRL 2999, ARS Culture Collection, Peoria, IL, USA) was routinely used. The *A. parasiticus* strain NFRI-95 is a norsolorinic acid (NA)-accumulating mutant isolated from *A. parasiticus* SYS-4, in which NA is a bright red-orange pigmental precursor of AFs [19]. *A. parasiticus* NFRI-26 is a mutant of *A. parasiticus* SYS-4 and produces neither AFs nor any pigmented precursors of AFs [19]. *Penicilium citrinum* NFRI-MI177 and NFRI-MI190 and *Chaetomium sphaerale* NFRI-MI163 and the unidentified strains NFRI-MI211 and NFRI-MI212 were isolated herein from soil or dead leaves. *Aspergillus oryzae* SYS-2 (IFO4251), a *Penicillium simplicissimum* strain, and a *Gibberella zeae* strain were also used [20].

### 2.2. Media

GY agar medium (2% glucose, 0.5% yeast extract, and 2% agar) was used for the visual agar plate assay as well as the microtiter agar plate assay [14]. YES (2% yeast extract and 20% sucrose), YEP (2% peptone and 0.5% yeast extract), GY (2% glucose and 0.5% yeast extract) liquid media, and Czapek Dox broth (CD, Difco Labs, Sparks, MD, USA) were used for the tip culture method [19]. Soyabean casein digest agar medium (SCD, Difco Labs) and potato dextrose agar medium (PDA, Difco Labs) were also used.

### 2.3. Detection of Microorganisms That are Inhibitors of Aflatoxin Production

#### 2.3.1. Visual Agar Plate Assay

For the selection of microorganisms that are inhibitors of aflatoxin production, microorganisms were isolated from environmental materials such as soil, living plants, and dead plants, and then were cultured on SCD plates. Then, each microorganism was picked up using a tooth pick and inoculated next to NFRI-95 at a distance of 1.5 cm on a 9 cm GY agar plate using the visual agar plate assay with minor modifications [14]. After 3–7 days of incubation at 28 °C, the effects of each microorganism on either the NA accumulation in the mycelium or the growth of NFRI-95 was visually observed from the underside of the plate. A decrease in the red pigment (NA) in the mycelium of the NFRI-95 strain indicated inhibition of aflatoxin production by the microorganism.

#### 2.3.2. Microtiter Agar Plate Assay

For the purification of substances that can inhibit the production of AFs, a small-scale assay system with a 96-well flat-bottom tissue culture plate (Microtest 96; Becton Dickinson, Franklin Lakes, NJ, USA) was used [14]. After the GY agar medium (100 μL) was solidified in each well of the culture plate, an aliquot (10–20 μL) of each fraction at each purification step of the inhibitory substance(s) was then applied onto the resulting GY agar in the well. Spore suspension of *A. parasiticus* NFRI-95 was inoculated onto the resulting agar medium and cultured at 28 °C for 2 or 3 days. The color of the fungal mycelia was observed from the underside of the plate. A decrease in the intensity of the NA red color or loss of the NA red color indicated that the tested fraction contained the inhibitory activity.

### 2.4. Identification of the Isolated Fungi

We performed a polymerase chain reaction (PCR) amplification of the region of 28S rDNA of the genome of the isolated fungus using primers LR0R and LR5 (http://www.biology.duke.edu/fungi/mycolab/primers.htm, accessed on 25 February 2016) [13]. The resulting PCR product was sequenced, and the sequence was analyzed by using a BLAST search.

### 2.5. Tip Culture Method

The tip culture method was used for the detection of fungal metabolites and for the measurement of the fungal growth [19]. A 1 mL Pipetman tip (Gilson, Middleton, WI, USA) was used as a culture vessel for the liquid stationary culture. First, 5 μL of spore suspension of each fungus was inoculated into 200 μL or 250 μL of liquid medium supplemented without or with CTN. After 4 days of incubation at 28 °C, the mycelium and culture medium were separated by centrifugation. For the detection of AFs, 10 μL of the medium was spotted onto a thin-layer chromatography (TLC) silica gel plate (silica gel 60 5721, Merck & Co., Whitehouse Station, NJ, USA), and then dried. The resulting plate was developed with either developing solution 1 (chloroform/ethyl acetate/90% formic acid (6:3:1, *v*/*v*/*v*)) or developing solution 2 (hexane/ethyl acetate/acetic acid (50:50:1, *v*/*v*/*v*)). The presence of AFs and CTN was evaluated under long-wavelength UV light (365 nm). For the detection of KA, the TLC plate was sprayed with 1% FeCl_3_ in 0.1 M HCl solution [21].

### 2.6. Feeding Experiment

Dihydrosterigmatocystin (DHST), a precursor of AFB_2_ and AFG_2_ in aflatoxin biosynthesis, was prepared using the hydrogenation of sterigmatocystin (ST) with 5% palladium charcoal [22]. For the examination of the in vivo conversion of DHST to AFs in aflatoxin biosynthesis, *A. parasiticus* strain NIAH-26 was cultured in YES medium supplemented with 40 μM DHST in the absence or presence of 0.105 mg mL^−1^ (0.42 mM) CTN at 28 °C for 4 days by using the tip culture method [23]. The amounts of AFB_2_ and AFG_2_ in the resulting medium were analyzed using a high-performance liquid chromatography (HPLC) apparatus (LCsolution, Shimadzu, Tokyo) equipped with a silica gel HPLC column (0.4 by 15 cm, Shim-pack CLC-SIL, Shimadzu) and a Shimadzu model RF-535 fluorescence detector (ex. 365 nm, em. 425 nm) and a solvent of toluene/ethyl acetate/formic acid (99%)/methanol (89:7.5:2:1.5, *v*/*v*/*v*) at a flow rate of 1.0 ml min^−1^ at 35 °C. A quantitative standard kit for AFs (B_1_, B_2_, G_1_, and G_2_) (Makor Chemicals, Jerusalem, Israel) was used for calibration.

### 2.7. RT-PCR

*A. parasiticus* strain SYS-4 was cultured by the tip culture method using 200 μL YES broth supplemented without or with 10 μg mL^−1^, 30 μg mL^−1^, and 105 μg mL^−1^ CTN at 28 °C for 4 days. The resulting mycelia were treated with TRI Reagent^®^ (Sigma-Aldrich, St. Louis, MO, USA) by using a FastPrep^®^ FP100A system (Q-BIO gene, Bio 101, Vista, CA, USA). The total RNA was prepared according to the manufacturer’s instructions (Sigma-Aldrich). After treatment with RNase-free DNase (Gibco BRL, Grand Island, NY, USA), reverse transcription (RT)-PCR using the total RNA was carried out using an RT-PCR kit (ReverTra Dash, Toyobo Co., Osaka, Japan). The primers used were: aflR-BamHI-F (CGCGGATCCATGGTTGACCATATCTCCCC) and aflRHindIII-R (CCCCAAGCTTCATTCTCGATGCAGGTAATC) for aflR (accession no. AF441437); HexB-F1 (CTGCGGGTGGAGCTGCA) and HexBR1 (CAAGCTCCAAGGGCGGC) for the hexanoate synthase gene (accession no. AF391094); pKSL1-F1 (CCAGGACAGCCCTATTCTAG) and pKSL1-R1 (GGAGTCCAGTGGTATTCAGC) for the polyketide synthetase gene; pksL1 (accession no. L42766), MT-1wholeF1 (ACAAATACCCCTGGCT CAGG), and MT-1wholeR1 (ACCTGTTCCATCAAATCGTC) for the *O*-methyltransferase I gene; and dmtA (accession no. AB022906) [14], CMDF1 (GATGGCCAGATCACCAC), and CMDR1 (CCGATGGAGGTCATGACG TG) for the calmodulin (*cmd*) gene (accession no. AY017584) [14].

### 2.8. Purification and Characterization

#### 2.8.1. Inhibitory Substance to Aflatoxin Biosynthesis (Substance A: CTN)

A substance that inhibits the biosynthesis of AFs (herein called substance A) was purified from the culture filtrate of the *P. citrinum* strain NFRI-MI190 by monitoring the inhibitory activity to the production of NA by strain NFRI-95. NFRI-MI190 was cultured in 100 mL of GY liquid medium at 28 °C for 4–7 days. The pH of the resulting culture filtrate was adjusted to pH 2.0–3.0 with 6 M HCl to change organic anions contained in the medium to their protonated forms, and then the resulting fungal metabolites in the filtrate were extracted with ethyl acetate. The ethyl acetate solution containing the inhibitory activity was extracted with 5% NaHCO_3_. Since the water phase contained the activity, the water-phase solution was acidified again to pH 2.0–3.0 with 6 M HCl, and then extracted with ethyl acetate.

The resulting ethyl acetate extract containing the inhibitory activity was applied onto a TLC plate (silica gel 60, Merck) and developed with the developing solution 2. A bright yellow fluorescent substance was observed under UV light (365 nm). The resulting plate was divided into five areas (a–e), and the substance(s) at each part were extracted with ethyl acetate and analyzed by a microtiter plate assay. The substance in the “c” part (Rf 0.37) (called substance A) showed inhibitory activity to the production of NA by strain NFRI-95.

For the characterization of substance A, we performed liquid chromatography-atmospheric pressure photoionization-mass spectrometry (LC-APPI-MS) with an LCMS-2010A apparatus (Shimadzu, Kyoto, Japan) equipped with a UV-Vis spectrophotometric detector (model SPD-6AV, Shimadzu), Ascentis C18 column (0.21 by 15 cm, Supelco). Absorption at 330 nm was monitored. The solvent system was 5 mM ammonium acetate buffer (pH 7.5) (solvent A) and methanol (solvent B); gradient: 10% solvent B was maintained for 2 min, changed linearly to 95% (2–17 min), and held for 5 min (17–22 min), followed by a return to the initial conditions (10% solvent B) within 1 min (22–23 min) and kept for 7 min (23–30 min) at the flow rate 0.2 mL min^−1^.

#### 2.8.2. The Substance Causing a Decrease in the Yellow Fluorescence of CTN (Substance B: KA)

The substance causing a decrease in the yellow fluorescence of CTN (called substance B) was purified from *A. parasiticus* NIAH-26 culture filtrate. The decrease in the yellow fluorescence of CTN was monitored by TLC as follows: Each fraction was incubated with CTN at 50 °C for 60 min, spotted on a silica gel TLC plate, and then developed with developing solution 2. The fluorescence of CTN was detected under UV light (365 nm).

For the purification of substance B, *A. parasiticus* NIAH-26 was cultured in YES liquid medium at 28 °C for 4 days, and then 4 mL of the culture filtrate was analyzed by TLC using a developing solution, *n*-buthanol/acetic acid/water (4:1:5, *v*/*v*/*v*). A light-brown band (Rf 0.6–0.7) on the TLC plate showed decreasing activity of CTN fluorescence. After substance B was extracted from the band with 50% methanol, the resulting extract was applied onto a Sep-Pak C18 cartridge (Waters). The activity was eluted with water, and the resulting water fraction was concentrated, and then purified with the HPLC apparatus equipped with a COSMOSIL 5C18-ODS column (Cosmosil 5C18-PQ, Nacalai Tesque, Kyoto, Japan) with monitoring at 270 nm. The solvent was 0.1% acetic acid and acetonitrile (10–95% gradient). Substance B was obtained as a single peak on the HPLC chromatogram.

#### 2.8.3. The Reaction Product Caused by the Incubation of CTN and Substance B (Substance C: CTN-KA Adduct)

CTN (2.7 mg or 3.7 mg) was incubated with 80 mM KA in 10 mM ammonium acetate buffer (pH 7.0) at 50 °C for > 18 h. After centrifugation at 13,000 rpm for 2 min, the supernatant was concentrated by using a centrifugal vaporizer to dryness. The residue was solubilized in a small volume of acetonitrile. The resulting solution was applied onto the HPLC apparatus equipped with an ODS column (Cosmosil 5C18-PQ) with a solvent of acetonitrile/water/TFA (250:750:0.5, *v*/*v*/*v*) at 1 mL min^−1^; absorption was monitored at 330 nm. The new peak eluted between KA and CTN corresponding to substance C was pooled and analyzed by physicochemical methods.

### 2.9. Physico-Chemical Analyses

Electron ionization-mass spectra (EI-MS) were recorded using a spectrometer (AX505HA, JEOL, Tokyo, direct probe, 70eV). Electrospray time-of-flight mass spectrometry (ESI-TOFMS) and high-resolution (HR)-ESI-TOFMS data were obtained with a mass spectrometer (LCT Premier XE, Waters, Milford, MA, USA). Nuclear magnetic resonance (NMR) spectra were measured with spectrometry (JNM-ECP 500, JEOL and Avance II 600, Bruker, Karlsruhe, Germany). Chemical shifts were referenced to acetone-*d*_6_ (δ_H_ 2.04 and δ_C_ 29.8).

## 3. Results

### 3.1. Isolation of Microorganisms That Inhibit the Production of AFs

**Figure 1:**
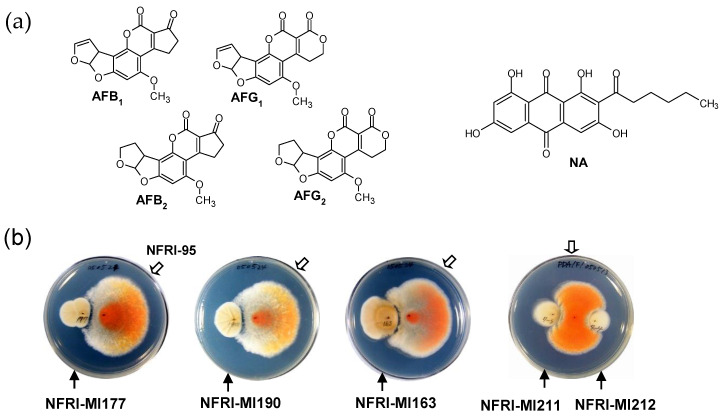
Inhibition of *A. parasiticus* strain NFRI-95’s accumulation of norsolorinic acid (NA) by isolated fungi: (**a**) Structures of the major AFs (AFB_1_, AFB_2_, AFG_1_, and AFG_2_) and NA, which has a bright red color; (**b**) the inhibition of strain NFRI-95’s NA production by isolated fungi. Spores of the isolated fungus (solid arrows) and spores of NFRI-95 (open arrows) were inoculated and cultured on the same plate. The accumulation of the red pigment (NA) of the NFRI-95 colony was examined from the underside of each plate. Fungi named NFRI-MI177, -MI190, and -MI163, but not NFRI-MI211 or -MI212, showed inhibitory effects on the production of NA by strain NFRI-95.

Since red pigmental NA is a precursor of AFs (Figure 1a), it was speculated that microorganisms that inhibit the production of NA may also inhibit the production of AFs. Thus, we used a visual plate assay with the NA-accumulating NIAH-95 strain to isolate microorganisms that inhibit aflatoxin production. We successfully isolated such microorganisms from environmental sources, i.e., soil, living plants, and dead plants at the National Food Research Institute (NARO), Japan. A total of 269 microorganisms (including 139 fungi) were obtained by using the visual agar plate assay (Figure 1b). Many of the microorganisms exerted an inhibitory effect on the production of NA and also on the growth of the fungus. Since the bacteria showed commonly weaker inhibitory activities as compared with those of the fungi, we investigated the fungi’s inhibition of NA production. Among the fungi, NFRI-MI190, NFRI-MI177, and NFRI-MI163 showed remarkable inhibition effects on the production of NA by NFRI-95 (Figure 1b). In contrast, other fungi such as NFRI-MI211 and NFRI-MI212 exerted little or no inhibition of NA production.

For the identification of the fungi NFRI-MI190, NFRI-MI177, and NFRI-MI163, a region of 28S rDNA of each was sequenced and analyzed by using a BLAST search. The PCR-amplified rDNA sequences of NFRI-MI190 and NFRI-MI177 showed 100% identity to that of *Penicillium citrinum* (accession no. AF484404) and *Aschersonia goldiana* (accession no. AY173424), respectively. The production of greenish conidia by these fungi and the typical morphology of conidiophores of these fungi supported that they belong to *Penicillium* spp. The rDNA sequence of NFRI-MI163 showed 100% identity with that of *Chaetomium sphaerale* (accession no. AF286407). The *P. citrinum* NFRI-MI190 strain was mainly used for further study herein.

### 3.2. The Substance Inhibiting the Biosynthesis of AFs

When we performed a small scale assay for inhibitory activity using microtiter agar plate assay, the filter-sterilized culture medium of the fungus NFRI-MI190 inhibited the accumulation of NA in strain NFRI-95. The inhibitory activity was not affected by heat treatment (121 °C, 15 min), which suggested that the inhibitory substance (which was called substance A) was a stable and low-molecular substance excreted from the mycelia.

**Figure 2:**
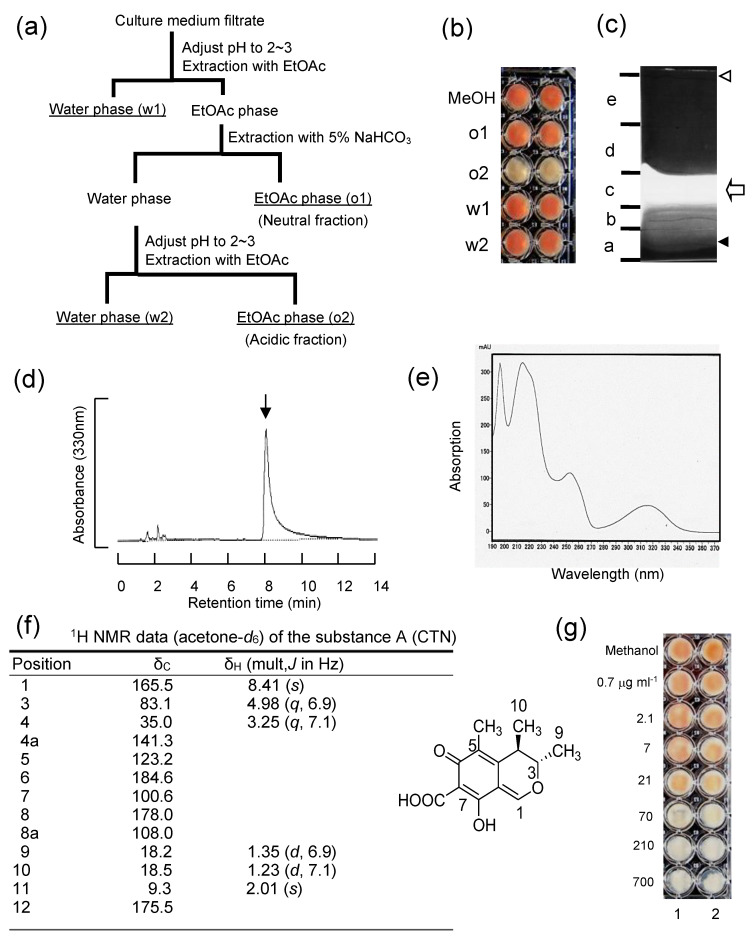
The purification of substance A from *P. citrinum* NFRI-MI190 culture medium: (**a**) The filtrate of the culture medium was fractionated at various conditions by organic solvent; (**b**) the inhibitory activity of each fraction (o1, o2, w1, and w2) was examined in a microtiter plate assay, showing that the inhibitory substance(s) was present in the o2 fraction; (**c**) the o2 fraction was analyzed by TLC. The inhibitory activity was observed at the c part, which showed bright yellow fluorescence (open arrow) (origin of the spot (closed triangle), front of the developing solution (open triangle)); liquid chromatography result (**d**) and the absorption spectrum (**e**) of the extract of the c part by the LC-APPI-MS analysis are shown; (**f**) the ^1^H NMR data (acetone-*d*_6_) and the structure of substance A; (**g**) microtiter plate assay using various concentrations of the purified substance A (lane 1) or the authentic standard of CTN (lane 2).

As the first step of purification of substance A, the culture filtrate of NFRI-MI190 was fractionated to four fractions (o1, o2, w1, and w2) by the solvent partitioning method (Figure 2a). The microtiter plate assays of each fraction revealed that substance A was fractionated in the o2 fraction (Figure 2b). Then the o2 fraction was analyzed by TLC and all of the area was divided into five parts (a–e) (Figure 2c). A substance (on the “c” part on the TLC plate) showed bright yellow fluorescence under UV light and the inhibitory activity was confirmed to be present at the “c” part. By using the tip culture method, it was also confirmed that the extract from the “c” part predominantly inhibited the production of AFs (data not shown). When the extract of the “c” part was subsequently analyzed by LC-APPI-MS, the extract showed a single peak on the HPLC chromatogram, and its retention time was the same as the authentic standard of CTN (no. 229580050, Thermo Fisher Scientific, Waltham, MA, USA) (Figure 2d). The molecular weight of the substance was 250, and the UV spectrum was identical to that of the authentic CTN (Figure 2e). The ^1^H- and ^13^C-NMR data of substance A showed agreement with the reported data of CTN (Figure 2f) [24,25]. The microtiter plate assay using various concentrations of substance A or the CTN standard showed the same CTN concentration dependency (Figure 2g). These results demonstrate that substance A is CTN.

### 3.3. The Mechanism Underlying the Inhibitory Effect of CTN on the Biosynthesis of AFs

**Figure 3:**
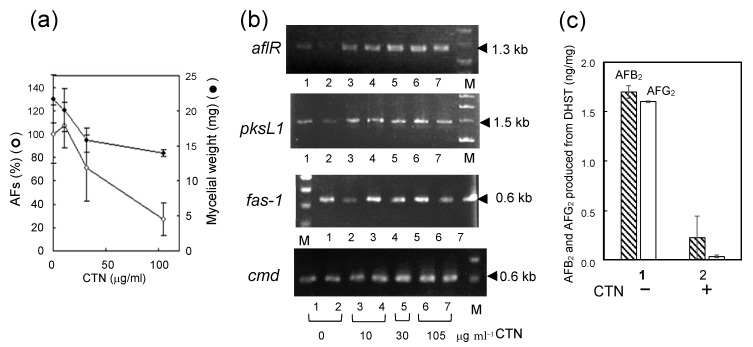
The effect of CTN on aflatoxin production, fungal growth, and gene expressions of aflatoxin biosynthesis genes: (**a**) The measurement of aflatoxin production and fungal growth by the tip culture method, the SYS-4 was cultured with various concentrations of CTN for 4 days, AFs in the resulting media were analyzed by HPLC, and the mycelial wet weight was measured, one hundred percent of the total AFs: 1.99 ± 0.24 μg mL^−1^; (**b**) the effects of CTN on the gene expression of aflatoxin biosynthesis genes, after the tip cultures of SYS-4 in (**a**), RNA fractions were prepared from the resulting mycelial mats, and RT-PCRs were then performed for aflatoxin biosynthetic genes (*aflR*, *pksL1*, and *fas-1*), and a housekeeping *cmd* gene; (**c**) the effect of CTN on the conversion of DHST to AFB_2_ as well as AFG_2_ in the feeding experiment, after *A. parasiticus* NIAH-26 was incubated with 40 μM DHST in the absence or the presence of 0.105 mg mL^−1^ CTN using tip culture method for 4 days, AFB_2_ and AFG_2_ in the resulting media were analyzed by HPLC, mycelial weights: lane 1, 17.6 ± 0.4 mg and lane 2, 13.7 ± 2.1 mg.

When *A. parasiticus* SYS-4 was incubated with various concentrations of CTN, aflatoxin production was remarkably inhibited at a CTN concentration of 30 μg mL^−1^ or more (Figure 3a). The inhibition of aflatoxin production was more remarkable than the inhibition of the growth of SYS-4. To investigate the mechanism underlying CTN’s inhibition of the production of AFs, RT-PCR was used to evaluate the effect of CTN on the expression of three types of aflatoxin genes (*aflR*, *pksL1*, and *fas-1*) and a housekeeping *cmd* gene (Figure 3b). The expressions of these genes were not affected even at a CTN concentration of 30 μg mL^−1^ or more. Since the *pksL1* and *fas-1* genes are involved in the production of NA in aflatoxin biosynthesis [7], these results indicated that the inhibition of NA production by CTN was not caused by inhibition of these gene expressions. In addition, since the *aflR* gene is a positive regulator of all enzyme genes involved in aflatoxin biosynthesis [7], these results indicated that CTN may have inhibited a certain post-transcriptional step in aflatoxin biosynthesis.

Then, the effect of CTN on in vivo aflatoxin production was examined by conducting a feeding experiment using the tip culture method. When *A. parasiticus* NIAH-26 was cultured with DHST, AFB_2_ and AFG_2_ were produced from DHST, because DHST is a precursor of these AFs (Figure 3c). However, when the same fungus was cultured with DHST in the presence of CTN, the amounts of AFB_2_ and AFG_2_ were greatly decreased. These results suggested that CTN inhibited a certain post-transcription step in aflatoxin biosynthesis.

### 3.4. Decrease in the Fluorescence of CTN by Aspergillus Species and the Inhibition of Fungal Growth by CTN

**Figure 4:**
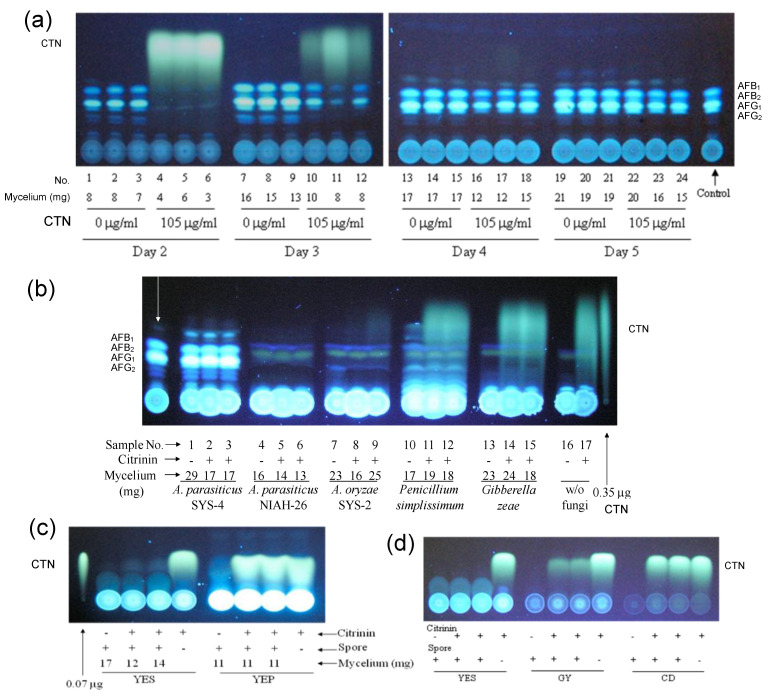
Disappearance of CTN fluorescence after incubation with various media: (**a**) *A. parasiticus* SYS-4 was cultured with 105 μg mL^−1^ of CTN in YES medium for 2–5 days, and the resulting culture media were analyzed by TLC, experiments were done in triplicate, developing solution 1 was used; (**b**) various species of fungi were cultured with CTN as in panel (**a**); (**c**) after *A. parasiticus* NIAH-26 was cultured with CTN in YES or YEP medium for 3 days by the tip culture method, the resulting medium was analyzed by TLC, mycelia weights are shown, developing solution 2 was used; (**d**) the same experiment as in panel (**c**) was done using YES, GY, or CD medium supplemented with CTN.

After *A. parasiticus* SYS-4 was incubated in YES medium supplemented without or with CTN for various numbers of days, the resulting culture media were analyzed by TLC (Figure 4a). The production of AFs was completely inhibited in the cultures with CTN at 2 days, and a significant amount of CTN was detected. At 3 days of incubation, the aflatoxin productivity of SYS-4 was partially recovered, whereas the amount of CTN was relatively decreased. At 4 and 5 days of incubation, the aflatoxin productivity was mostly recovered, whereas CTN had completely disappeared. These results suggested that (i) the inhibition of aflatoxin production with CTN was a transient reaction and (ii) CTN appeared to be an unstable substance that disappeared after several days. Regarding the fungal growth, the mycelial weight of SYS-4 was partially, but significantly, decreased with CTN (Figure 4a).

Growth inhibition with CTN and the disappearance of CTN were also observed when the aflatoxin-non-producing *A. parasiticus* strain NIAH-26 and *A. oryzae* SYS-2 were cultured with CTN for 4 days (Figure 4b). In contrast, these phenomena were not detected when *P. simplicissimum* and *G. zeae* were used. Together, these results suggested that the growth inhibition with CTN as well as the disappearance of CTN may be species-specific phenomena.

*A. parasiticus* strain NIAH-26 was incubated with CTN in YES medium or YEP medium (Figure 4c). CTN was not decreased when YEP instead of YES was used, and the decreasing extent of CTN was less or much less when GY or CD medium was used (Figure 4d). We suspected that a metabolite (which was called substance B) was related to the decrease in the yellow fluorescence of CTN and that substance B would be produced depending on the culture medium. The pattern of the dependency was very similar to that of the aflatoxin production by aflatoxigenic fungi.

### 3.5. Identification of the Substance Causing a Decrease in the Yellow Fluorescence of CTN

For the characterization of substance B, a four-day culture medium of NIAH-26 was filter-sterilized, and then heated at 95 °C for 15 min. When the resulting medium was incubated with CTN, the resulting medium still had the activity to decrease CTN fluorescence. These results suggested that substance B was a heat-stable substance excreted from the cells.

**Figure 5:**
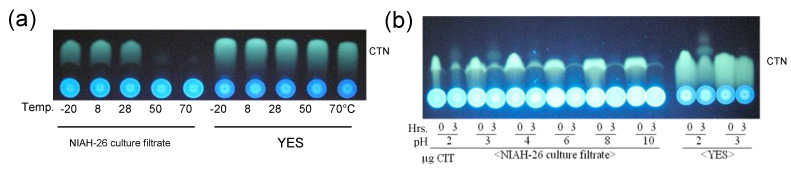
The disappearance of CTN fluorescence by filter-sterilized culture medium: (**a**) The culture medium of NIAH-26 was sterilized by filter filtration, the resulting culture filtrate was incubated with CTN at various temperatures for 3 h, and the resulting medium was analyzed by TLC; (**b**) the sterilized medium filtrate of NIAH-26 or YES medium was adjusted to various pH values, and then added with 105 μg mL^−1^ CTN, and then incubated at 50 °C for 3 h, and the resulting media were analyzed by TLC using developing solution 2.

When the filter-sterilized culture medium was incubated with CTN at various temperatures for 3 h (Figure 5a), the CTN was decreased at 50 °C and 70 °C, whereas the use of YES medium did not show a decrease in the yellow fluorescence of CTN, indicating that a higher temperature accelerated the decrease in CTN fluorescence by substance B. The pH dependency of the decrease in CTN fluorescence was also examined at 50 °C for 3 h (Figure 5b). The CTN was significantly decreased at all pH conditions used. However, we used ammonium acetate buffer (pH 7.0) or potassium phosphate buffer (pH 7.5) to ensure the reproducibility of the results in the subsequent experiments.

**Figure 6:**
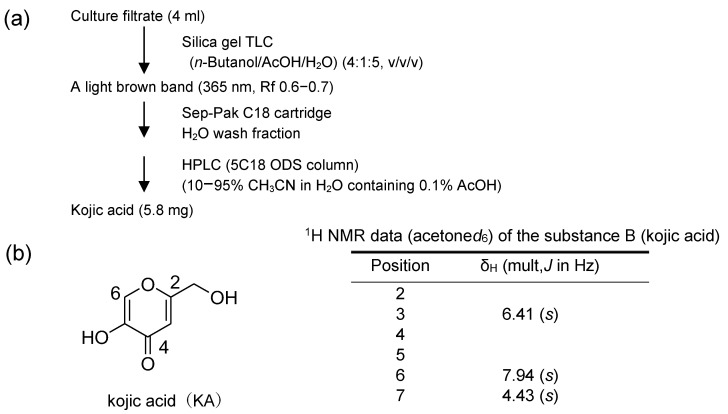
The purification and clarification of the structure of substance B that decreased the CTN fluorescence: (**a**) Purification steps of substance B from the culture medium of NIAH-26; (**b**) ^1^H NMR data of substance B. Substance B was confirmed to be KA.

Purification of substance B was performed from the culture filtrate of NIAH-26 using successive chromatographies: TLC twice, Sep-Pak C18 column chromatography, and HPLC (Figure 6a). As a result, 5.8 mg of substance B was obtained from 4 mL of the culture filtrate of NIAH-26. The EI-MS and ^1^H-NMR analyses showed that substance B was KA (Figure 6b).

### 3.6. The Formation of the CTN-KA Adduct

**Figure 7:**
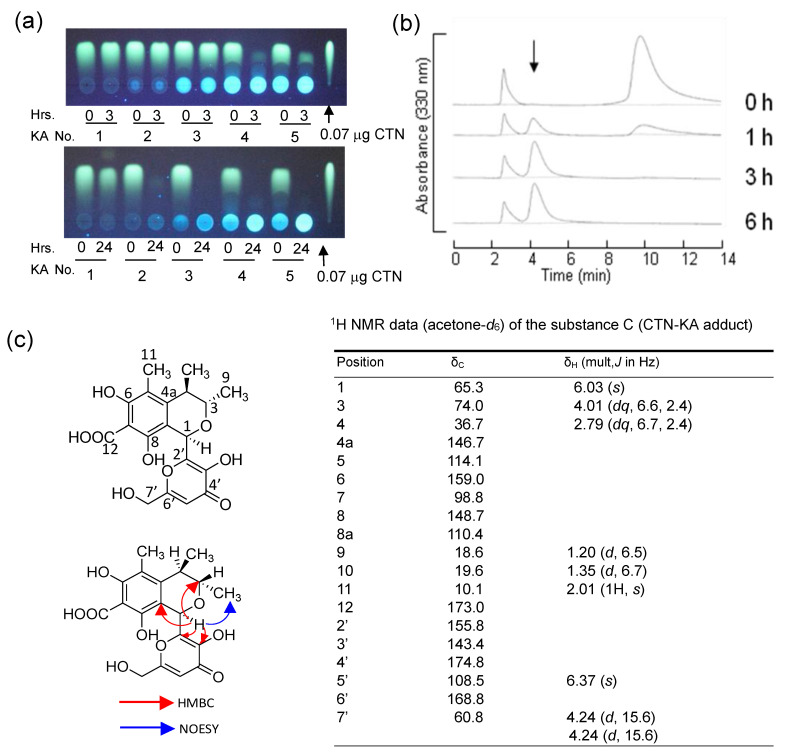
The formation of the CTN-KA adduct and the confirmation of its structure: (**a**) CTN fluorescence decrease by KA. CTN (1.6 × 10^−2^ mmol) was incubated with various concentrations of KA (lane 1, no KA; lane 2, 1.6 × 10^−2^ mmol KA; lane 3, 1.6 × 10^−1^ mmol KA; lane 4, 1.6 mmol KA; lane 5, the purified substance from NIAH-26 in 42 mL of 48 mM potassium phosphate buffer (pH 7.5) at 50 °C for 3 h (*upper* TLC plate) or 24 h (*lower*). The resulting solutions (15 mL each) were analyzed by TLC using developing solution 2; (**b**) the time course of the detection of the reaction product (*arrow*). Reaction mixtures containing 2 mM CTN and 75 mM KA were incubated at 50 °C for the indicated periods and analyzed by HPLC using an ODS column and the solvent acetonitrile/water/TFA (450:550:0.5, *v*/*v*/*v*) and monitoring at 330 nm; (**c**) the structure of the novel substance produced from CTN and KA was determined by NMR. Correlations obtained by heteronuclear multiple bond correlation (HMBC) and nuclear Overhauser effect spectroscopy (NOESY) in substance C are shown.

To clarify what happened during the decrease in the yellow fluorescence of CTN, various concentrations of KA were incubated with 1.6 × 10^−2^ mmol of CTN at 50 °C for 3 or 24 h (Figure 7a). Although 3 h of incubation was not enough to completely decrease 1.6 × 10^−2^ mmol CTN with the same concentration (1.6 × 10^−2^ mmol) of KA, the 24 h incubation caused the complete disappearance of CTN with 1.6 × 10^−2^ mmol KA at a one-to-one ratio (Figure 7a, lower panel, lane 2). The addition of more KA also caused the complete disappearance of CTN. Even with 3 h of incubation, CTN mostly disappeared with a 100-fold concentration of KA (upper panel, lane 4). These results indicated that CTN reacted with KA at a one-to-one ratio and that when a higher concentration of KA as compared with CTN was used, the decrease in the yellow fluorescence of CTN occurred faster.

To investigate the reaction product of CTN’s disappearance with KA, CTN and KA were incubated at 50 °C for various periods, and the resulting metabolites were analyzed by HPLC. After 1 h of incubation, a novel peak appeared at ~4.5 min and increased with time, and at the same time, the peaks of CTN and KA decreased (Figure 7b). These results indicated that a novel substance (which we called substance C) was produced from CTN and KA.

For the identification of the structure of substance C, CTN was incubated with KA and the resulting substance C was purified by preparative HPLC, and then analyzed by physico-chemical methods (Figure 7c). The ESI-TOFMS analysis of substance C revealed a positive ion peak at *m*/*z* 393 and a negative ion peak at *m*/*z* 391, indicating that the molecular weight was 392. The molecular formula of the reaction product was determined to be C_19_H_20_O_9_ from the high-resolution ESI-TOFMS data (calcd for C_19_H_20_O_9_H^+^, 393.1186 and found 393.1187 (Figure S1), and calcd for C_19_H_19_O_9_^−^, 391.1029 and found 391.1047 (Figure S2)) and ^13^C NMR data (Figure S3). The resonances observed in the spectra of KA and CTN were almost detected in the ^1^H NMR spectrum of substance C (Figure S4); however, the resonance assigned to the hydrogen at C-6 of KA (δ_H_ 7.94) disappeared and the resonance assigned to the hydrogen bonded to C-1 of CTN was significantly shifted from δ_H_ 8.41 to δ_H_ 6.03. In light of these results, substance C was presumed to be an adduct of CTN and KA, not a CTN decomposition product.

Based on the ^13^C NMR (Figure S3), heteronuclear multiple-quantum correlation (HMQC, Figure S5), and -bond correlation (HMBC, Figure S6) data for substance C, the signals assignable to C-1 (δ_C_ 165.5), C-6 (δ_C_ 184.6), and C-8 (δ_C_ 178.0) in CTN shifted to δ_C_ 65.3, δ_C_ 159.0, and δ_C_ 148.7 in substance C, respectively. The HMBC analysis also revealed a correlation from the hydrogen at C-1 of the CTN portion to C-6 and C-5 of the KA portion. These facts and other COSY (Correlation Spectroscopy), HMQC, and HMBC correlations clarify the structure of substance C in which CTN is bonded to KA through C-1 of CTN and C-6 of KA. The C-1 sp^2^ carbon of CTN changes into sp^3^ carbon; the left ring containing a keto group of CTN is aromatized, and the keto group at the C-6 changes into a phenolic hydroxyl group. Thus, substance C was identified as 3,4-dihydro-6,8-dihydroxy-1-(3-hydroxy-6-(hydroxymethyl)-4-oxo-4*H*-pyran-2-yl)-3,4,5-trimethyl-1*H*-isochromene-7-carboxylic acid. The NOESY examination (Figure S7) detected a correlation between the hydrogen at C-1 and the hydrogens of C-9 methyl, indicating that the hydrogen at C-1 and the C-9 methyl have the same orientation with respect to the ring. Since the absolute configuration of C-3 and C-4 of CTN was determined, the stereochemistry of substance C was determined to be 1*R*, 3*S*, 4*R* (Figure 7c). These results demonstrated that the substance was (1*R*,3*S*,4*R*)-3,4-dihydro-6,8-dihydroxy-1-(3-hydroxy-6-(hydroxymethyl)-4-oxo-4*H*-pyran-2-yl)-3,4,5-trimethyl-1*H*-isochromene-7-carboxylic acid, which was herein named the ”CTN-KA adduct”.

**Figure 8:**
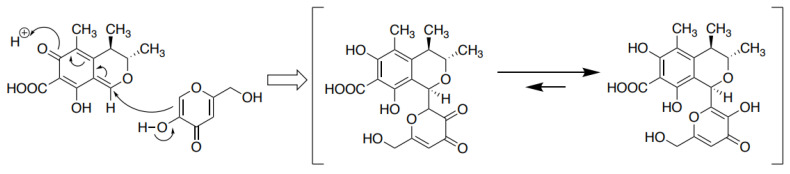
A proposed reaction mechanism for the formation of the CTN-KA adduct.

We propose the following reaction mechanism for the formation of the CTN-KA adduct (Figure 8). The π electrons of C-6 in the KA molecule attack C-1 of CTN, resulting in bond formation between two molecules, followed by a double bond transition, eventually aromatizing the ring on the left side of CTN. KA attacks CTN from the β-side of the CTN ring, since the C-9 methyl on the α side of CTN hinders an attack of KA from the α-side. Although the CTN-KA adduct theoretically exists as two possible structures, the enol-type structure (right side in the bracket) may exist much more than the ketone-type one (left side) based on their structural stabilities. In fact, physico-chemical analyses of CTN-KA adduct demonstrated that the adduct was mostly an enol-type structure (Figure 7c).

### 3.7. Stability of the CTN-KA Adduct

**Figure 9:**
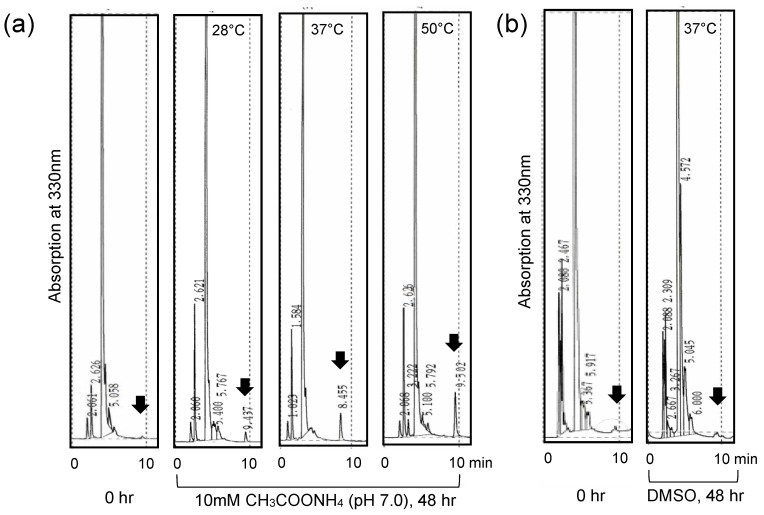
Stability of the CTN-KA adduct: (**a**) CTN-KA adduct was incubated in 10 mM ammonium acetate buffer (pH 7.0) at 28 °C, 37 °C, or 50 °C for 48 h. The resulting metabolites were analyzed by HPLC using ODS columns and the solvent acetonitrile/water/TFA (450:550:0.5, *v*/*v*/*v*) at 1 mL min^−1^. The peaks of CTN (black thick arrows) increased with the temperature in this aqueous solution; (**b**) CTN-KA adduct was incubated in DMSO at 37 °C for 48 h, CTN did not increase, suggesting that the CTN-KA adduct was stable in DMSO.

To confirm the stability of the CTN-KA adduct, the adduct was incubated in 10 mM ammonium acetate buffer (pH 7.0) at various temperatures for 48 h. The HPLC analysis of the resulting substance showed that approximately 0.3% of the CTN-KA adduct was converted to CTN at 28 °C (Figure 9a). Incubation of the adduct at 37 °C and 50 °C caused conversions of 1.0% and 1.3% of the adduct to CTN, respectively. In contrast, when the adduct was incubated in DMSO at 37 °C for 48 h, conversion was not detected (Figure 9b). These results indicated that the CTN-KA adduct was considerably stable in aqueous condition even at 50 °C, and that it was also stable in DMSO.

### 3.8. Effect of the CTN-KA Adduct on the Production of AFs

**Figure 10:**
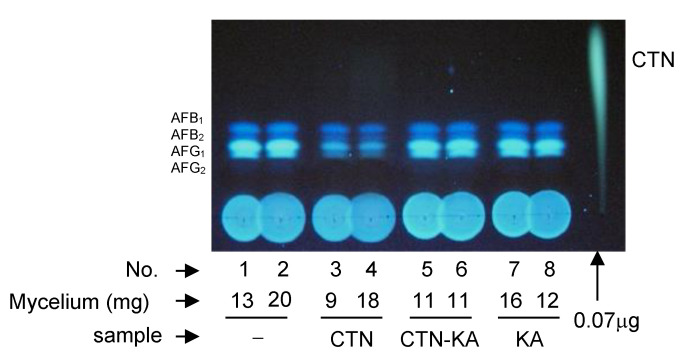
The effects of CTN, the CTN-KA adduct, and KA on the production of AFs in the tip culture. Spore suspension (5 μL) of *A. parasiticus* strain SYS-4 was added to 200 μL YES medium supplemented with CTN, the CTN-KA adduct, or KA, and then cultured for 4 days. For the evaluation of the effect of the adduct, 1.9 mM CTN and 71.4 mM KA were pre-incubated in 50 mM K-phosphate buffer (pH 7.5) at 50 °C for 6 h. The resulting solution (40 μL) was then added to 160 μL YES medium in a tip culture, and the spore suspension was added and then cultured. After culture at 28 °C for 4 days, the culture media were analyzed by TLC using developing solution 1. Mycelial weights are also shown. The experiments were done in duplicate.

To investigate the inhibitory activity of the CTN-KA adduct on the production of AFs, the *A. parasiticus* strain SYS-4 was incubated with each of CTN, CTN-KA adduct, and KA by the tip culture method (Figure 10). The CTN-KA adduct (as well as KA) did not inhibit aflatoxin production, whereas CTN significantly inhibited the production. These results indicated that the toxicity of CTN was detoxified by conversion to the CTN-KA adduct. 

## 4. Discussion

### 4.1. CTN’s Inhibition of Aflatoxin Production and Fungal Growth

The results of this study demonstrated that (i) CTN inhibited the production of AFs by *A. parasiticus* and (ii) higher concentrations of CTN also inhibited growth of the fungus (Figure 3a and Figure 4a). CTN is a mycotoxin produced mainly by strains of the genera *Penicillium*, *Monascus*, and *Aspergillus*. CTN contamination has been reported in cereals (maize, wheat, rice, barley, and oats) and cereal-based products, pomaceous fruits and fruit juice, roasted nuts, cheese, and more [16,17,18]. *Monascus* species have been used in the food industry for the production of fermented foods, edible red pigments, and monacolin K, which is a cholesterol synthesis inhibitory substance. The so-called “moldy rice poisoning” cases that occurred in Japan in 1953–1954 were also due to CTN contamination by these fungi [26]. CTN has been reported to be a causative substance of nephropathy and hepatotoxicity as well as renal adenoma formation in various mammalian cell types and animals [16,17,18,27]. Antibiotic activities of CTN against many types of microorganisms including fungi have also been described [28,29]. The present study provides the first report that a mycotoxin, CTN, inhibited the production of another mycotoxin, AFs. Since CTN and AFs are, respectively, produced by different fungi belonging to different fungal genera, i.e., *Penicillium* and *Aspergillus* fungus, we speculated that the different fungi might interact via secondary metabolites produced by both fungi.

This work showed that CTN did not inhibit the expression of aflatoxin biosynthesis genes, indicating that CTN may not inhibit the transcription step of the aflatoxin biosynthesis genes. However, CTN remarkably inhibited the conversion of DHST to AFB_2_ and AFG_2_ in the feeding experiment (Figure 3c), suggesting that CTN may have affected one or more post-transcriptional steps in the biosynthesis of AFs. CTN is known to exert a variety of toxic effects in animals and humans through oxidative stress and mitochondrial dysfunction [30,31,32]. Haraguchi et al. reported that approximately half of the CTN taken up was found in mitochondria and that CTN inhibited succinate oxidase and NADH oxidase [33]. Regarding CTN’s toxicity to the kidney and liver, the inhibition site of CTN has been speculated to be the electron transport system of mitochondria [27]. An examination of mRNA expression profiles in *Saccharomyces cerevisiae* has shown that the oxidative stress response genes were significantly induced by CTN [28]. Therefore, oxidative stress caused by CTN may be involved in the inhibition of aflatoxin production as well as growth inhibition. Detailed studies of this possibility are in progress in our laboratory.

### 4.2. Formation of CTN-KA Adduct

The present analyses demonstrated that CTN was bound to KA to form a novel CTN-KA adduct, in which a covalent C-C bond was non-enzymatically formed between C-1 of CTN and C-6 of KA (Figure 7). Because the resulting CTN-KA adduct did not have fluorescence, the decrease in the yellow fluorescence of CTN in the culture medium of *A. parasiticus* at 28 °C was used as an indicator of the conversion of CTN to the CTN-KA adduct. It has been reported that to detoxify CTN, heat treatment at ~130–150 °C converted CTN to various substances, and some of them were more toxic than CTN [34,35]. In our present investigation, a much lower temperature such as 50 °C or 70 °C was sufficient for the formation of the CTN-KA adduct. Although the conversion rate to the adduct was faster at 50 °C than at 28 °C, the conversion occurred for 24 h even at 28 °C. In addition, the reaction product was only the CTN-KA adduct; no by-product was obtained (Figure 7). Equilibrium of the reaction was inclined toward the formation of the adduct in aqueous conditions (Figure 8). 

Our examination of the stability of the CTN-KA adduct revealed that a slight amount (0.3–1.3%) of CTN was reproduced after incubation of the CTN-KA adduct from 28 °C to 50 °C for 48 h (Figure 9). Since the food-safety regulated value of CTN is relatively high [36], the slight amount of CTN possibly reproduced from the adduct will not become a great concern. Therefore, the conversion of CTN to CTN-KA with KA could be used as a safe method for the detoxification of CTN. 

Kojic acid is well known in the cosmetic industry as a tyrosinase inhibitor, and extensive studies of the development of stronger tyrosinase inhibitors than KA have been performed. Noh et al. developed KA-amino acid conjugates and observed that KA-phenylalanine amide (KA-F-NH(2)) had strong inhibitory activity [37]. Morteza et al. produced several KA-phenolic natural products by using phenolic substances such as umbelliferon, sesame, thymol, and apocynin [38]. However, since these conjugates were produced by conjugation between each substance and KA using click reaction and 1,2,3-triazole formation, they are different from the CTN-KA substance described herein. Poór et al. reported that CTN formed a stable complex with human serum albumin, and H-bonds, salt bridges, and hydrophobic interactions were involved as the binding force [39]. These were different from the covalent binding. The present experiments demonstrated that CTN bound to KA via a covalent C-C bond at a one-to-one ratio (Figure 8). The CTN-KA adduct is a novel substance. Since KA is known as a chelate reagent, KA adduct formation might be useful for producing other types of adducts.

CTN is known to be a nephrotoxic and hepatotoxic substance to humans and animals and to be an antibiotic, antifungal, anti-bacteriophage, anti-sarcoma, anti-protozoa, and anti-animal cells substance [16,17,18]. This work showed that unlike CTN, the CTN-KA adduct did not inhibit the production of AFs by *A. parasiticus*, suggesting that the adduct’s formation could be a useful detoxification method of CTN. In the future, we aim to examine the toxicity of the CTN-KA adduct to bacteria and culture cells, and then to investigate the function of secondary metabolites in fungal interactions.

## Data Availability

The data presented in this study are available in supplementary material here.

## Supplementary Materials

The following are available online at https://www.mdpi.com/article/10.3390/jof9010029/s1, Figure S1, High-resolution (HR)-ESI-TOFMS spectrum of CTN-KA adduct (positive ion mode); Figure S2, High-resolution (HR)-ESI-TOFMS spectrum of CTN-KA adduct (negative ion mode); Figure S3, 13C-NMR spectrum of CTN-KA adduct (Acetone-d6); Figure S4, 1H-NMR spectrum of CTN-KA adduct (Acetone-d6); Figure S5 HMQC spectrum of CTN-KA adduct (Acetone-d6); Figure S6 HMBC spectrum of CTN-KA ad-duct (Acetone-d6); Figure S7 NOESY spectrum of CTN-KA adduct (Acetone-d6).

## References

[1] Tola M., Kebede B. (2016). Occurrence, Importance and Control of Mycotoxins: A Review. Cogent Food Agric..

[2] Food and Agriculture Organization of the United Nations (2004). Mycotoxin Regulations in 2003 and Current Developments. Worldwide Regulations for Mycotoxins in Food and Feed.

[3] Higginson J., DeVita V.T. (1980). IARC Monographs on the Evaluation of Carcinogenic Risk of Chemicals to Humans. Am. Ind. Hyg. Assoc. J..

[4] Williams J.H., Phillips T.D., Jolly P.E., Stiles J.K., Jolly C.M., Aggarwal D. (2004). Human Aflatoxin in Developing Countries: A Review of Toxicology, Exposure, Potential Health Consequences, and Interventions. Am. Soc. Clin. Nutr..

[5] Wu F., Liu Y., Bhatnagar D. (2008). Cost-Effectiveness of Aflatoxin Control Methods: Economic Incentives. Toxin Rev..

[6] Bennett J.W., Klich M. (2003). Mycotoxins. Clin. Microbiol. Rev..

[7] Yu J. (2012). Current Understanding on Aflatoxin Biosynthesis and Future Perspective in Reducing Aflatoxin Contamination. Toxins.

[8] Yabe K., Chihaya N., Hatabayashi H., Kito M., Hoshino S., Zeng H., Cai J., Nakajima H. (2012). Production of M-/GM-Group Aflatoxins Catalyzed by the OrdA Enzyme in Aflatoxin Biosynthesis. Fungal Genet. Biol..

[9] Ehrlich K.C. (2014). Non-Aflatoxigenic *Aspergillus flavus* to Prevent Aflatoxin Contamination in Crops: Advantages and Limitations. Front. Microbiol..

[10] Yoshinari T., Akiyama T., Nakamura K., Kondo T., Takahashi Y., Muraoka Y., Nonomura Y., Nagasawa H., Sakuda S. (2007). Dioctatin A Is a Strong Inhibitor of Aflatoxin Production by *Aspergillus parasiticus*. Microbiology.

[11] Rajani P., Sridevi V., Lakshmi M.V.V.C. (2012). A Review on Biological Control of Aflatoxin Crop Contamination. Int. J. Chem. Environ. Pharm. Res..

[12] Abbas H.K., Zablotowicz R.M., Bruns H.A., Abel C.A. (2006). Biocontrol of Aflatoxin in Corn by Inoculation with Non-Aflatoxigenic *Aspergillus flavus* Isolates. Biocontrol Sci. Technol..

[13] Yabe K., Yan P.S., Song Y., Ichinomiya M., Nakagawa H., Shima Y., Ando Y., Sakuno E., Nakajima H. (2008). Isolation of Microorganisms and Substances Inhibitory to Aflatoxin Production. Food Addit. Contam..

[14] Yan P., Song Y., Sakuno E., Nakajima H. (2004). Cyclo (l-Leucyl-l-Prolyl) Produced by *Achromobacter xylosoxidans* Inhibits Aflatoxin Production by *Aspergillus parasiticus*. Appl. Environ. Microbiol..

[15] Iimura K., Furukawa T., Yamamoto T., Negishi L., Suzuki M., Sakuda S. (2017). The Mode of Action of Cyclo(L-Ala-L-Pro) in Inhibiting Aflatoxin Production of *Aspergillus flavus*. Toxins.

[16] Kamle M., Mahato D.K., Gupta A., Pandhi S., Sharma B., Sharma N., Sharma B., Mishra S., Arora S., Selvakumar R. (2022). Citrinin Mycotoxin Contamination in Food and Feed: Impact on Agriculture, Human Health, and Detection and Management Strategies. Toxins.

[17] Silva L.J.G., Pereira A.M.P.T., Pena A., Lino C.M. (2021). Citrinin in Foods and Supplements: A Review of Occurrence and Analytical Methodologies. Foods.

[18] Doughari J.H. (2015). The Occurrence, Properties and Significance of Citrinin Mycotoxin. J. Plant Pathol. Microbiol..

[19] Yabe K., Nakamura H., Ando Y., Terakado N., Nakajima H., Hamasaki T. (1988). Isolation and Characterization of *Aspergillus parasiticus* Mutants with Impaired Aflatoxin Production by a Novel Tip Culture Method. Appl. Environ. Microbiol..

[20] Cai J., Zeng H., Shima Y., Hatabayashi H., Nakagawa H., Ito Y., Adachi Y., Nakajima H., Yabe K. (2008). Involvement of the NadA Gene in Formation of G-Group Aflatoxins in *Aspergillus parasiticus*. Fungal Genet. Biol..

[21] Bentley R. (1957). Preparation and Analysis of Kojic Acid. Methods Enzymol..

[22] Davies J.E., Kirkaldy D., Roberts J.C. (1960). 437. Studies in Mycological Chemistry. Part VII. Sterigmatocystin, a Metabolite of *Aspergillus versicolor*(Vuillemin) Tiraboschi. J. Chem. Soc..

[23] Yabe K., Ando Y., Hamasaki T. (1988). Biosynthetic Relationship among Aflatoxins B_1_, B_2_, G_1_, and G_2_. Appl. Environ. Microbiol..

[24] Sankawa U., Ebizuka Y., Noguchi H., Isikawa Y., Kitaghawa S., Yamamoto Y., Kobayashi T., Iitak Y., Seto H. (1983). Biosynthesis of Citrinin in *Aspergillus Terreus*. Tetrahedron.

[25] Poupko R., Luz Z., Destro R. (1997). Carbon-13 NMR of Citrinin in the Solid State and in Solutions. J. Phys. Chem. A.

[26] Kushiro M. (2015). Historical Review of Researches on Yellow Rice and Mycotoxigenic Fungi Adherent to Rice in Japan. Mycotoxins.

[27] Flajs D., Peraica M. (2009). Toxicological Properties of Citrinin. Arh. Hig. Rada Toksikol..

[28] Iwahashi H., Kitagawa E., Suzuki Y., Ueda Y., Ishizawa Y., Nobumasa H., Kuboki Y., Hosoda H., Iwahashi Y. (2007). Evaluation of Toxicity of the Mycotoxin Citrinin Using Yeast ORF DNA Microarray and Oligo DNA Microarray. BMC Genom..

[29] Leite M.C.A., De Brito Bezerra A.P., De Sousa J.P., Guerra F.Q.S., De Oliveira Lima E. (2014). Evaluation of Antifungal Activity and Mechanism of Action of Citral against *Candida albicans*. Evid.-Based Complement. Altern. Med..

[30] Kumar R., Dwivedi P.D., Dhawan A., Das M., Ansari K.M. (2011). Citrinin-Generated Reactive Oxygen Species Cause Cell Cycle Arrest Leading to Apoptosis via the Intrinsic Mitochondrial Pathway in Mouse Skin. Toxicol. Sci..

[31] Chen C.C., Chan W.H. (2009). Inhibition of Citrinin-Induced Apoptotic Biochemical Signaling in Human Hepatoma G2 Cells by Resveratrol. Int. J. Mol. Sci..

[32] Da Lozzo E.J., Oliveira M.B.M., Carnieri E.G.S. (1998). Citrinin-Induced Mitochondrial Permeability Transition. J. Biochem. Mol. Toxicol..

[33] Haraguchi H., Tanaka T., Taniguchi M., Oi S., Hashimoto K. (1986). Inhibitory Effects of Citrinin on Mitochondrial Function. Agric. Biol. Chem..

[34] Trivedi A., Doi E., Kitabatake N. (1993). Toxic Compounds Formed on Prolonged Heating of Citrinin under Watery Conditions. J. Food Sci..

[35] Hirota M., Menta A.B., Yoneyama K., Kitabatake N. (2002). A Major Decomposition Product, Citrinin H2, from Citrinin on Heating with Moisture. Biosci. Biotechnol. Biochem..

[36] Commission Regulation (EU) 2019/1901 of 7 November 2019 Amending Regulation (EC) No 1881/2006 as Regards Maximum Levels of Citrinin in Food Supplements Based on Rice Fermented with Red Yeast Monascus purpureus. https://eur-lex.europa.eu/legal-content/EN/TXT/PDF/?uri=CELEX:32019R1901&from=EN.

[37] Pillaiyar T., Manickam M., Namasivayam V. (2017). Skin Whitening Agents: Medicinal Chemistry Perspective of Tyrosinase Inhibitors. J. Enzyme Inhib. Med. Chem..

[38] Ashooriha M., Khoshneviszadeh M., Khoshneviszadeh M., Rafiei A., Kardan M., Yazdian-Robati R., Emami S. (2020). Kojic Acid–Natural Product Conjugates as Mushroom Tyrosinase Inhibitors. Eur. J. Med. Chem..

[39] Poór M., Lemli B., Bálint M., Hetényi C., Sali N., Kőszegi T., Kunsági-Máté S. (2015). Interaction of Citrinin with Human Serum Albumin. Toxins.

