# Supplementary material for: Inhibition of Aflatoxin Production by Citrinin and Non-Enzymatic Formation of a Novel Citrinin-Kojic Acid Adduct
Source: J Fungi (Basel). 2022 Dec 23;9(1):29. doi: 10.3390/jof9010029 (PMC9861921; doi:10.3390/jof9010029)
Supplement: Supplementary file 1 [file jof-09-00029-s001.zip › jof-2011569-supplementary.pdf]

Supplement Materials

Inhibition of Aflatoxin Production by Citrinin and Non-Enzymatic Formation of a Novel Citrinin-Kojic Acid Adduct

Ichinomiya, M.; Fukushima-Sakuno, E.; Kawamoto, A.; Nakagawa, H.; Hatabayashi, H.; Nakajima, H.; Yabe, K.

Figure S1. High-resolution (HR)-ESI-TOFMS spectrum of CTN-KA adduct (positive ion mode).

Figure S2. High-resolution (HR)-ESI-TOFMS spectrum of CTN-KA adduct (negative ion mode).

Figure S3. ^{13}C-NMR spectrum of CTN-KA adduct (Acetone-d_6).

Figure S4. ^{1}H-NMR spectrum of CTN-KA adduct (Acetone-d_6).

Figure S5. HMQC spectrum of CTN-KA adduct (Acetone-d_6).

Figure S6. HMBC spectrum of CTN-KA adduct (Acetone-d_6).

Figure S7. NOESY spectrum of CTN-KA adduct (Acetone-d_6).

Single Mass Analysis

Tolerance = 5.0 PPM / DBE: min = -1.5, max = 50.0
Element prediction: Off
Number of isotope peaks used for i-FIT = 3

Monoisotopic Mass, Even Electron Ions
199 formula(e) evaluated with 2 results within limits (up to 50 best isotopic matches for each mass)
Elements Used:
C: 1-30 H: 1-40 N: 0-5 O: 1-10
120315004 38 (1.156)
1: TOF MS ES+

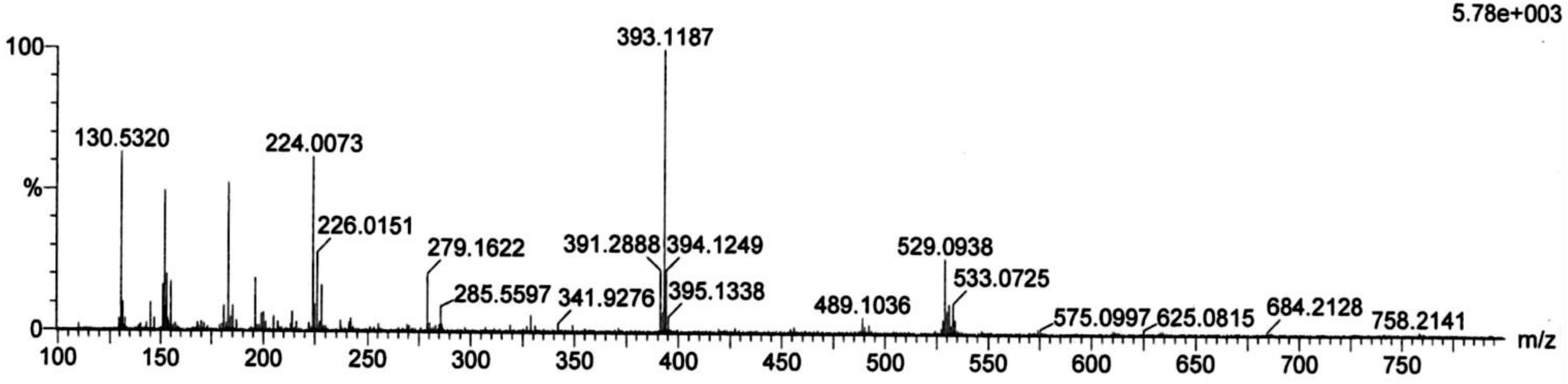

Minimum:				-1.5			
Maximum:		100.0	5.0	50.0			
Mass	Calc. Mass	mDa	PPM	DBE	i-FIT	i-FIT (Norm)	Formula
393.1187	393.1186	0.1	0.3	9.5	76.5	0.2	C19 H21 O9
	393.1199	-1.2	-3.1	14.5	78.1	1.7	C20 H17 N4 O5

Figure S1. High-resolution (HR)-ESI-TOFMS spectrum of CTN-KA adduct (positive ion mode).

Single Mass Analysis
Tolerance = 10.0 PPM / DBE: min = -1.5, max = 50.0
Element prediction: Off
Number of isotope peaks used for i-FIT = 3

Monoisotopic Mass, Even Electron Ions
199 formula(e) evaluated with 3 results within limits (up to 50 best isotopic matches for each mass)
Elements Used:
C: 1-30 H: 1-40 N: 0-5 O: 1-10
120315004 64 (1.952)
2: TOF MS ES-

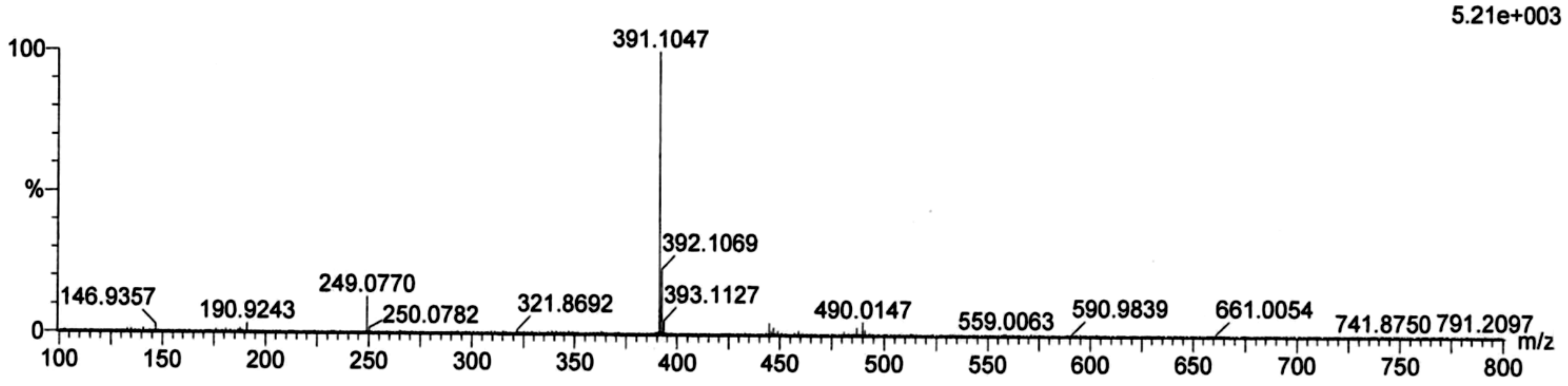

Minimum:				-1.5
Maximum:		100.0	10.0	50.0

Mass	Calc. Mass	mDa	PPM	DBE	i-FIT	i-FIT (Norm)	Formula
391.1047	391.1029	1.8	4.6	10.5	64.1	0.6	C19 H19 O9
	391.1042	0.5	1.3	15.5	64.4	0.9	C20 H15 N4 O5
	391.1083	-3.6	-9.2	19.5	70.3	6.7	C25 H15 N2 O3

Figure S2. High-resolution (HR)-ESI-TOFMS spectrum of CTN-KA adduct (negative ion mode).

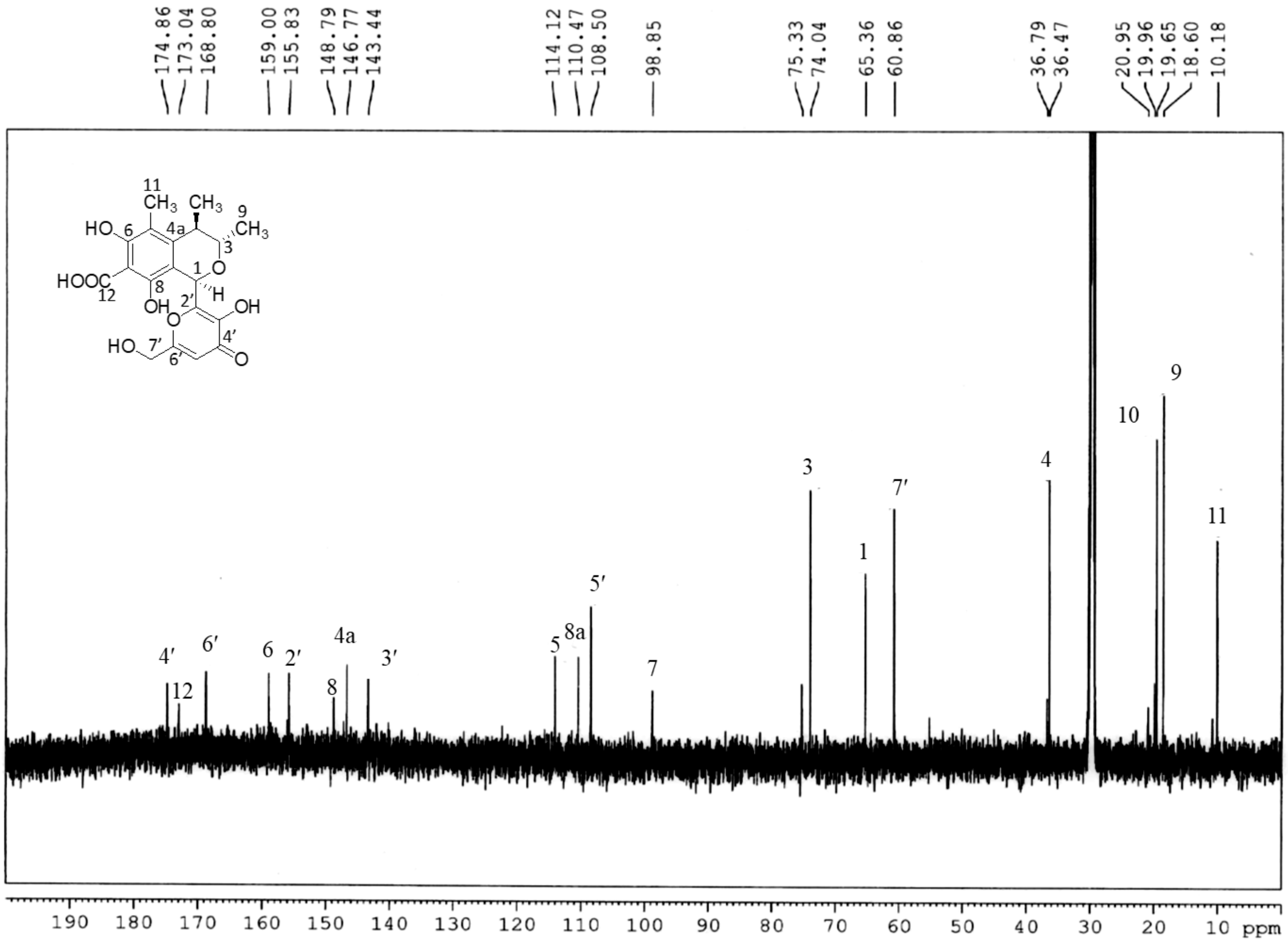

Figure S3. ^{13}C-NMR spectrum of CTN-KA adduct (Acetone-d_6).

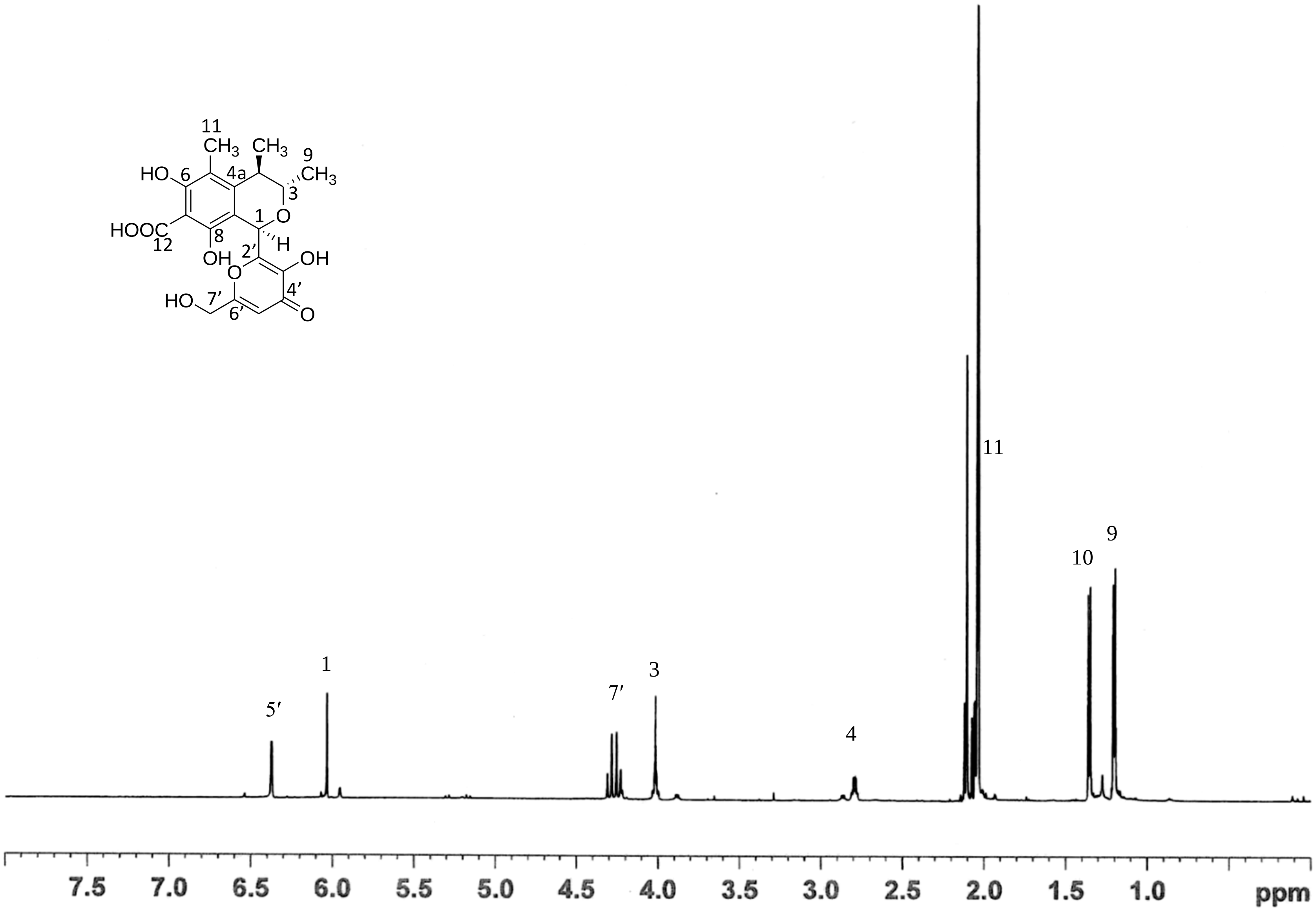

Figure S4. ^{1}H-NMR spectrum of CTN-KA adduct (Acetone-d_6).

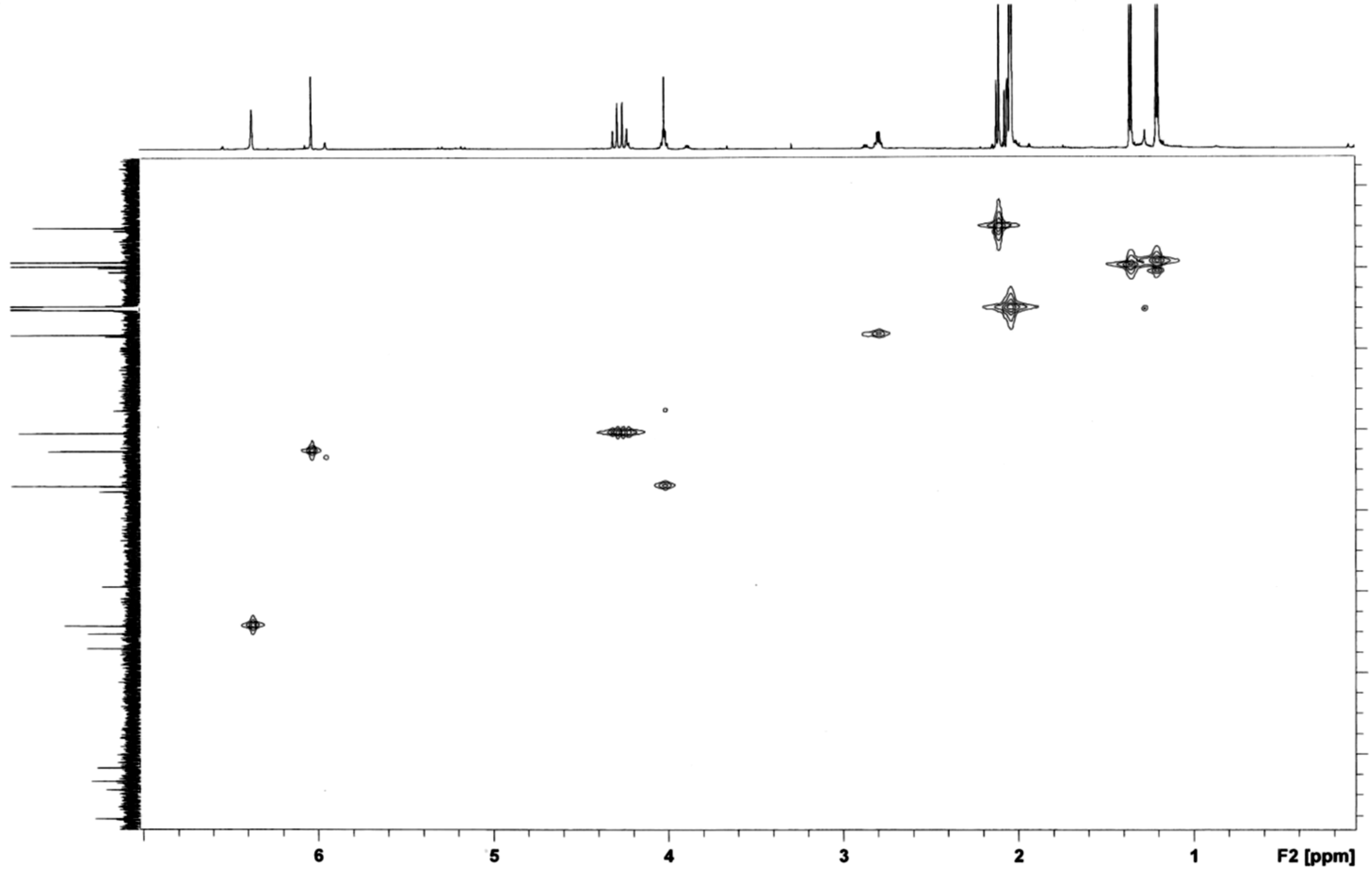

Figure S5. HMQC spectrum of CTN-KA adduct (Acetone-d_6).

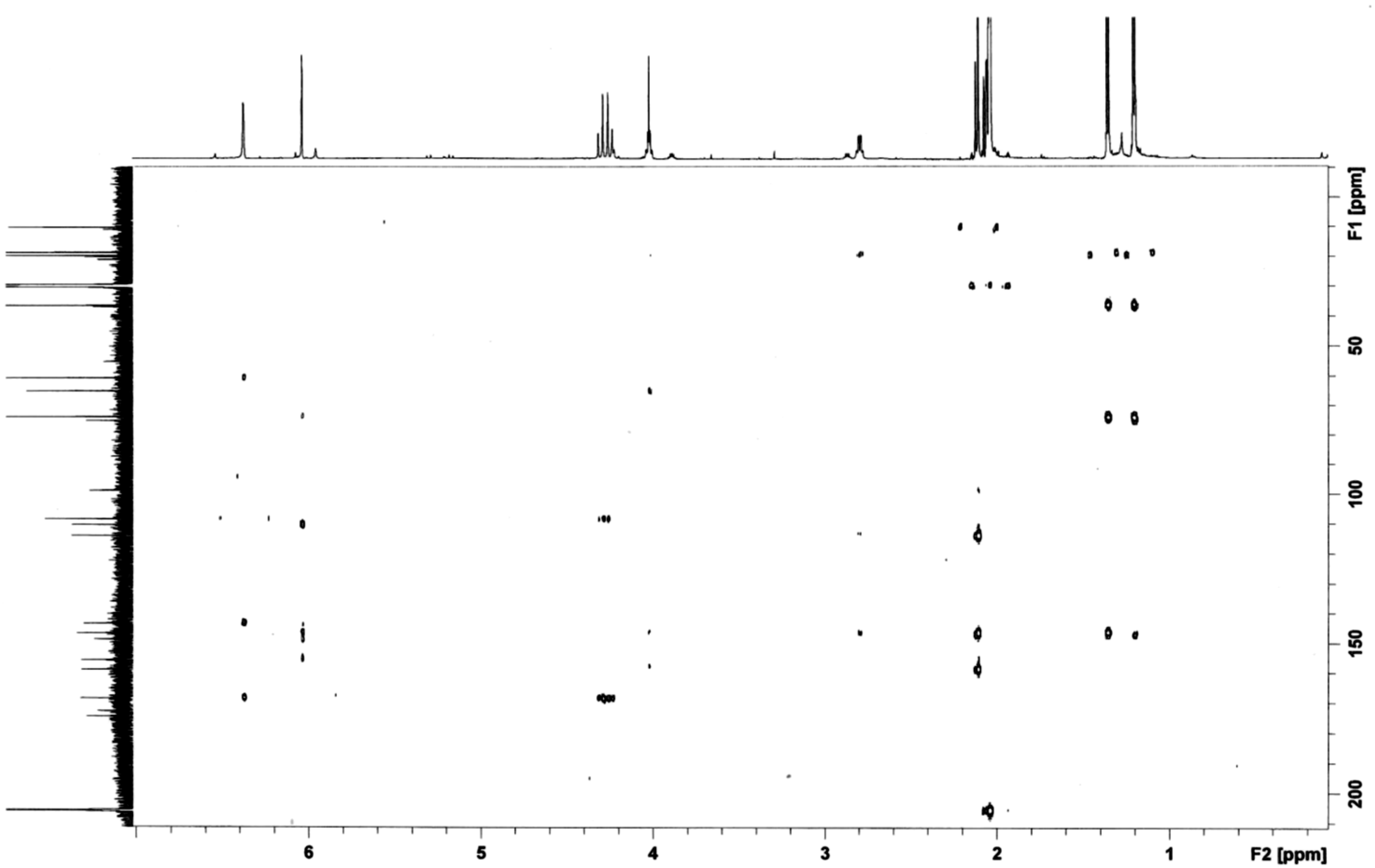

Figure S6. HMBC spectrum of CTN-KA adduct (Acetone-d_6).

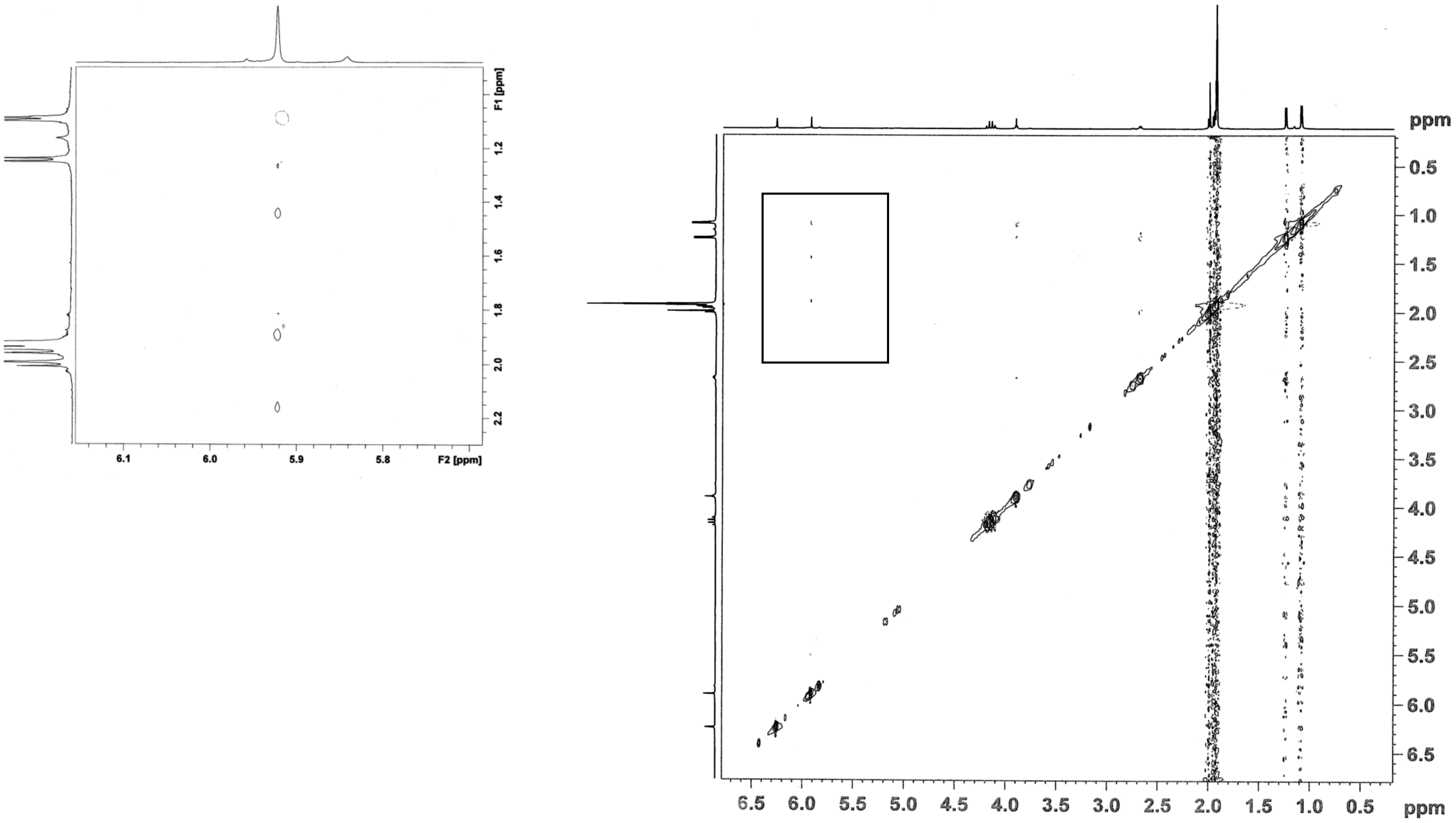

Figure S7. NOESY spectrum of CTN-KA adduct (Acetone-d_6).
